# Genetics of Peripartum Cardiomyopathy: Current Knowledge, Future Directions and Clinical Implications

**DOI:** 10.3390/genes12010103

**Published:** 2021-01-15

**Authors:** Timothy F. Spracklen, Graham Chakafana, Peter J. Schwartz, Maria-Christina Kotta, Gasnat Shaboodien, Ntobeko A. B. Ntusi, Karen Sliwa

**Affiliations:** 1 Hatter Institute for Cardiovascular Research in Africa & CHI, Department of Medicine, Faculty of Health Sciences, University of Cape Town, Cape Town 7935, South Africa; sprtim002@myuct.ac.za (T.F.S.); graham.chakafana@uct.ac.za (G.C.); p.schwartz@auxologico.it (P.J.S.); gasnat.shaboodien@uct.ac.za (G.S.); ntobeko.ntusi@uct.ac.za (N.A.B.N.); 2 Division of Cardiology, Department of Medicine, Groote Schuur Hospital, Faculty of Health Sciences, University of Cape Town, Cape Town 7925, South Africa; 3 Center for Cardiac Arrhythmias of Genetic Origin and Laboratory of Cardiovascular Genetics, Istituto Auxologico Italiano, IRCCS, 20135 Milan, Italy; m.kotta@auxologico.it; 4 Cape Universities Body Imaging Centre, Faculty of Health Sciences, University of Cape Town, Cape Town 7925, South Africa

**Keywords:** peripartum cardiomyopathy, genetic cardiomyopathy, heat shock proteins, chaperones

## Abstract

Peripartum cardiomyopathy (PPCM) is a condition in which heart failure and systolic dysfunction occur late in pregnancy or within months following delivery. Over the last decade, genetic advances in heritable cardiomyopathy have provided new insights into the role of genetics in PPCM. In this review, we summarise current knowledge of the genetics of PPCM and potential avenues for further research, including the role of molecular chaperone mutations in PPCM. Evidence supporting a genetic basis for PPCM has emanated from observations of familial disease, overlap with familial dilated cardiomyopathy, and sequencing studies of PPCM cohorts. Approximately 20% of PPCM patients screened for cardiomyopathy genes have an identified pathogenic mutation, with *TTN* truncations most commonly implicated. As a stress-associated condition, PPCM may be modulated by molecular chaperones such as heat shock proteins (Hsps). Recent studies have led to the identification of Hsp mutations in a PPCM model, suggesting that variation in these stress-response genes may contribute to PPCM pathogenesis. Although some Hsp genes have been implicated in dilated cardiomyopathy, their roles in PPCM remain to be determined. Additional areas of future investigation may include the delineation of genotype-phenotype correlations and the screening of newly-identified cardiomyopathy genes for their roles in PPCM. Nevertheless, these findings suggest that the construction of a family history may be advised in the management of PPCM and that genetic testing should be considered. A better understanding of the genetics of PPCM holds the potential to improve treatment, prognosis, and family management.

## 1. Introduction

Peripartum cardiomyopathy (PPCM) is a rare form of cardiac muscle disease associated with pregnancy. PPCM is a potentially lethal condition which is defined as heart failure (HF) and left ventricular systolic dysfunction occurring late in pregnancy or within months following delivery and in the absence of other causes of HF [1]. Although left ventricular recovery is possible up to 12 months following diagnosis, adverse outcomes such as irreversible HF, arrhythmia and sudden cardiac death are frequent [2,3].

The reported incidence of PPCM varies greatly between population groups, from approximately 1:1000 pregnancies in South Africa [4] to 1:10,000 pregnancies in Denmark [5]. Higher incidence of PPCM in Nigeria, South Africa and African Americans reported historically suggests that individuals of African descent may be at greater risk of PPCM [4,6]. However, a multicentre registry spanning 43 countries indicated that PPCM is a global disease that is likely to be under-reported in many instances and can occur in women of any ethnic background [7]. Other documented PPCM risk factors include multiparity, increased maternal age, and family history of cardiovascular disease [1]. The disparity in PPCM prevalence across various populations suggests that apart from environmental factors, a strong genetic basis for the condition may exist.

**Figure 1:**
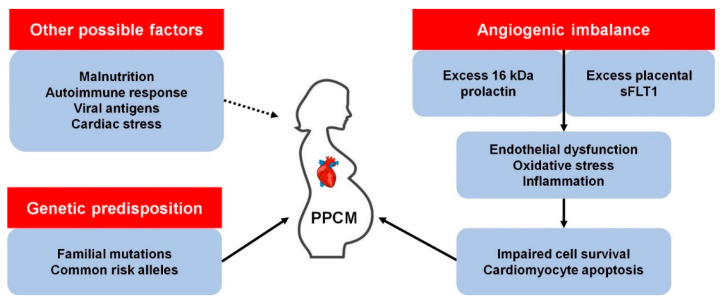
Known and possible mechanisms of peripartum cardiomyopathy (PPCM) pathophysiology. *kDa, kilodalton; sFLT1, soluble fms-like tyrosine kinase-1.*

Precisely how PPCM develops is unclear, although there are several proposed mechanisms (Figure 1). It has been observed that pregnancy induces dramatic haemodynamic changes, such as reduced afterload and an increase in cardiac output and blood volume [8]. These changes trigger homeostatic and structural remodelling of cardiovascular tissues which results in exacerbated cardiac stress. As such, the hormonal changes associated with parturition may trigger endothelial dysfunction and PPCM in susceptible women [9]. Over the last decade, much progress has been made towards understanding the genetics of familial forms of cardiomyopathy such as dilated cardiomyopathy (DCM). Given the role of family history and genetic factors in PPCM, these advances have afforded opportunities to better understand the genetics of PPCM. This review summarises current knowledge of the genetic contribution to PPCM and highlights potential avenues for future research.

## 2. The Role of Familial Cardiomyopathy Genes in PPCM

**Figure 2:**
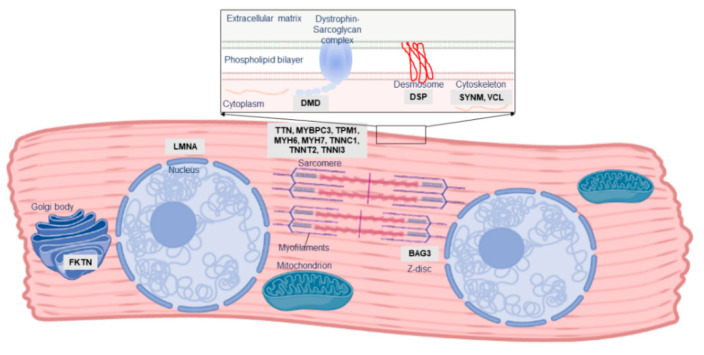
Localisation and function of PPCM-associated genes in the cardiomyocyte. The majority of PPCM genes, including Titin (*TTN*), comprise the cardiac sarcomere, but mutations affecting numerous other aspects of cardiomyocyte function have been implicated in PPCM pathogenesis.

One of the first indications of the role of genetic susceptibility in PPCM pathogenesis was familial clustering of PPCM with other forms of cardiomyopathy. Three instances of familial PPCM were reported in 1963 by Pierce et al. [10]; many similar familial occurrences of PPCM and DCM have been subsequently documented [11,12,13,14,15,16,17]. Although PPCM is a distinct clinical entity, these observations of overlap with familial DCM indicate that, in at least a subset of patients, PPCM may form part of the clinical and genetic spectrum of DCM. DCM itself is a vastly heterogeneous disease, genetically overlapping with hypertrophic cardiomyopathy (HCM), arrhythmogenic cardiomyopathy (ACM), and channelopathies: mutations in many of these genes have also been described in PPCM patients (Table 1; Figure 2).

In what appears to be the first documented genetic cause of PPCM, a 2001 case report described a female carrier of a Duchenne Muscular Dystrophy-causing *DMD* mutation who developed PPCM during the 36th week of pregnancy [18]. As an X-linked gene, most female carriers of *DMD* mutations are asymptomatic although some may develop mild disease or cardiomyopathy, and it was unclear if the PPCM, in this case, was related to her carrier status. However, similar case reports of PPCM amongst *DMD* and *LAMP2* X-linked mutation carriers have been described [19,20,21]. *LAMP2* mutations cause Danon disease, a form of cardiomyopathy and skeletal myopathy, through impairment of macroautophagy [22], while *DMD* mutations cause muscular dystrophy and/or DCM through the loss of the stabilising protein dystrophin, leaving myocytes vulnerable to oxidative stress and calcium overload [23]. In both cases, it is thought that skewed X-chromosome inactivation, in which only some cells express the *DMD* or *LAMP2* mutation, underlies the comparatively milder cardiac phenotype observed in females [24]. It is possible, too, that this mosaic status in females may be exacerbated by stress conditions such as pregnancy, although no evidence of this has been reported as yet.

*DMD* and *LAMP2* mutations are very rare causes of DCM; notably, most known DCM-causing mutations affect the sarcomere, causing cardiomyopathy through impaired force generation or reduced contractility in the heart. Early investigations reported several mutations in the sarcomeric genes *MYBPC3*, *MYH6*, *MYH7*, *TNNC1* and *TNNT2* in Dutch and American PPCM cohorts [13,17,25]. As such, similar studies across several other populations may provide critical information into the role of these mutations in PPCM.

As knowledge of DCM genetics has evolved over time, so too has knowledge of the genetic contribution to PPCM. Subsequent to these initial investigations, the giant sarcomeric protein-coding gene *TTN* emerged as a key gene in cardiomyopathy, accounting for up to 25% of familial DCM cases [26]. As the second-longest gene in humans, genetic variation in *TTN* is common [27], and it may be challenging to differentiate benign polymorphisms from true cardiomyopathy-causing mutations. However, frameshift insertions/deletions and nonsense mutations (truncating mutations) in *TTN* have been reported at an increased prevalence in DCM patients [26,27]. The mechanisms underlying *TTN* cardiomyopathy are thought to involve impairment of force generation or transmission in the myocardium or disrupted signalling [28]. *BAG3* has also recently emerged as a major cardiomyopathy gene [29]. *BAG3* encodes a multifunction co-chaperone protein involved in myocardial protein homeostasis, stabilisation of the myocardial Z disk, and modulation of cardiac contraction [30]. Mutations in *BAG3* can cause cardiomyopathy through impairments in any of the protein’s diverse functions [31].

**Figure 3:**
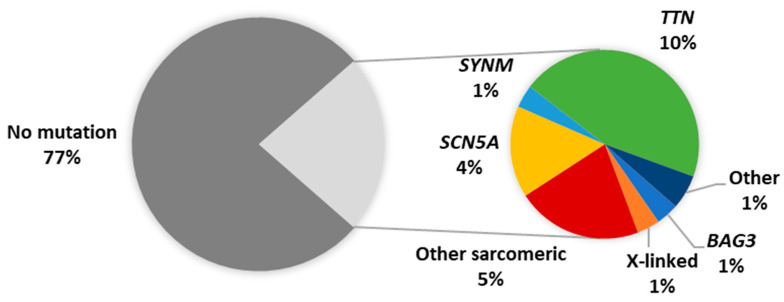
Proportion of reported PPCM patients with pathogenic mutations. Other sarcomeric genes: *MYBPC3*, *MYH6*, *MYH7*, *TNNC1*, *TNNT2*, *TPM1*; X-linked genes: *DMD*, *LAMP2*; Other genes: *DSP*, *PSEN2*, *VCL*.

The emergence of high throughput next-generation sequencing (NGS) platforms enabled the rapid sequencing of patient genes and has highlighted the genetic overlap between different forms of heritable cardiomyopathy. Following these genetic advances, NGS studies of PPCM cohorts have revealed truncating mutations in *TTN* as the predominant genetic contributor to PPCM in American, Australian and European patients [32,33,34]. Although such *TTN* mutations are present in healthy individuals, they were found to be significantly more prevalent amongst PPCM patients [33]. In addition, the *TTN* mutations were mostly localised to the protein domains already associated with DCM (A-band), thereby implicating their possible role in PPCM [33]. In these NGS studies, mutations were reported in other cardiac disease genes, including disruptions of *BAG3* [32,33,34]. Cumulatively, these cohort studies of PPCM have identified pathogenic mutations in 23% of cases (Figure 3). Studies of isolated PPCM cases have further implicated *TTN* in severe PPCM [35], as well as other disease genes such as *KCNH2*, *RET* and *TXNRD2* [16,36,37], although the contribution of these genes to PPCM is unclear: the mutations in these cases may merely reflect the co-occurrence of genetic disorders with PPCM. Mutations in *FKTN*, *RBM20*, *LMNA* and *DSP* were also linked to PPCM through large cardiomyopathy family studies [38,39,40,41].

The diverse genetic aetiology of PPCM (Table 1) mirrors the genetic profile of DCM and strengthens the notion that PPCM may share genetic determinants with familial cardiomyopathy. In PPCM, as in DCM, a high proportion of *TTN* truncating variants is observed, and at present appears to be the greatest single genetic contributor to disease pathogenesis [32,33]. The mutation detection rate of genetic testing in DCM ranges from 15% to 40%, with familial history and larger gene panels contributing to higher mutation yields [42]. Although within this range, the relatively low yield (~20%) in PPCM may be attributed to the substantially fewer patients who have been screened so far, combined with fewer genes being sequenced in earlier studies.

**Figure 4:**
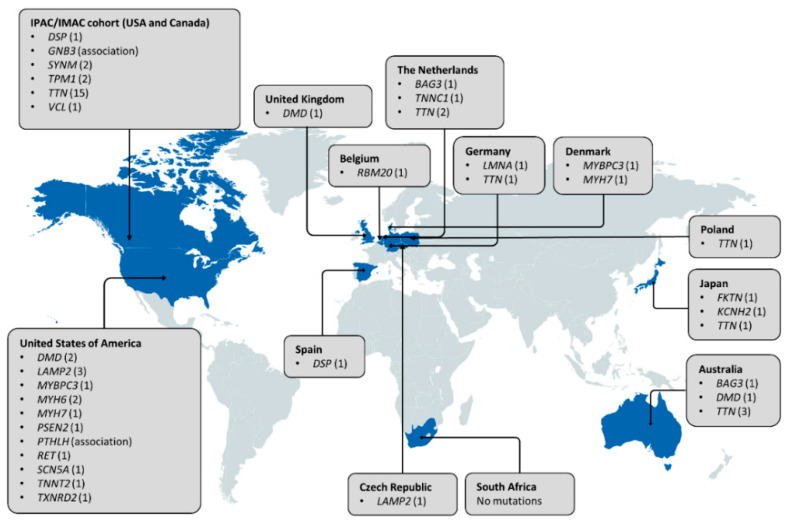
Distribution of genes associated with PPCM to date. Countries in which genetic investigations of PPCM patient(s) have been conducted are indicated in blue. The number of pathogenic mutations identified in each gene is indicated in parentheses. Three pathogenic *MYH7* mutations and seven *SCN5A* mutations are not included as the countries of origin could not be determined.

When considering the worldwide distribution of PPCM-associated mutations (Figure 4), it is noteworthy that the majority have been identified in American and European populations, but even then, many of the genes are limited to single observations and case reports. Analysis of participants of African descent by Ware et al. [33] revealed mutation yields that were akin to those in individuals of European ancestry, indicating a similar genetic basis of PPCM in different population groups. This also suggests that mutation screening of African PPCM cohorts may be warranted. In DCM, the clinical utility of genetic testing can extend to family management and individual treatment and prognosis, through the characterisation of genotype-phenotype correlations. For example, *LMNA* mutations are associated with severe DCM with high penetrance and increased risk of sudden death, while *TTN* mutations have been associated with milder disease with reduced penetrance [43]. Mutations in these genes have been reported in PPCM, but their implications on phenotype and outcome are still to be determined. Further research is also needed to determine whether the co-occurrence of cardiomyopathy mutations in PPCM patients reflects causality or if, rather, it is the physiological strain of pregnancy that reveals cardiomyopathy in previously asymptomatic mutation carriers.

## 3. Other Genetic Determinants of PPCM

As a complex disorder, the role of genetics in PPCM may not be limited to rare, high-impact mutations as observed in familial cardiomyopathy. PPCM may also be influenced by more common variants with smaller effects on individual susceptibility or outcome: such variants are best identified through genetic association studies. In a 2011 genome-wide association study of 41 PPCM cases and 49 healthy controls, Horne et al. [44] investigated common genetic variation for associations with PPCM. Despite the small sample size, *PTHLH* rs258415 reached statistical significance, and its association with PPCM was replicated in a second cohort of 30 cases and 124 controls. *PTHLH* has documented cardiovascular roles including modulation of ventricular contraction and regulation of blood flow in the placenta and uterus. The authors hypothesised that the gene may be upregulated in pregnancy to prevent uterine contraction, and variation in *PTHLH* could compromise heart function and predispose individuals to PPCM [44].

In an outcomes-based genetic analysis, Sheppard et al. [45] revealed that the *GNB3* c.825C>T polymorphism, specifically the homozygous TT allele, was significantly associated with poorer outcomes, with lower ejection fractions up to a year postpartum compared to individuals without the variant. The TT allele was also determined to be more prevalent in patients of African ethnicity. The gene may play a role in hypertension and cardiac remodelling, although its role in PPCM remains to be confirmed.

## 4. An Emerging Field: Heat Shock Protein and Molecular Chaperone Genes in PPCM

The aforementioned studies indicate a clear role of genetics in PPCM pathophysiology. Nevertheless, the 23% mutation yield from the screening of cardiomyopathy genes (Figure 3) suggests that many genetic determinants of PPCM are yet to be discovered. Indeed, many of the reported PPCM/DCM families had no identified mutation [13,32]. There is, therefore, a need to consider other genes for their contribution to PPCM. Given the role of cardiac stress in the development of the disease, molecular chaperones and heat shock proteins (Hsps) may prove of interest due to their stress-protective functions.

Lending support to this notion is the prior identification of *BAG3* mutations in PPCM patients [32,34]. As a molecular chaperone, BAG3 is actively involved in a variety of cellular mechanisms during stress which includes protein folding, autophagy, and the ubiquitin-proteasome system [46]. BAG3 exerts some of these functions by acting as a co-chaperone for Hsp70 and the small Hsps (sHsps) HspB5, HspB6 and HspB8 [47]. The formation of the BAG3-Hsp70-sHsp complex is essential in facilitating denatured proteins to refold thus preventing protein aggregation in cell stress conditions. Because *BAG3* is a key cardiomyopathy gene, with several reported DCM-causing mutations occurring across the gene, many more causative variants in the gene are likely still to be discovered amongst PPCM patients.

**Figure 5:**
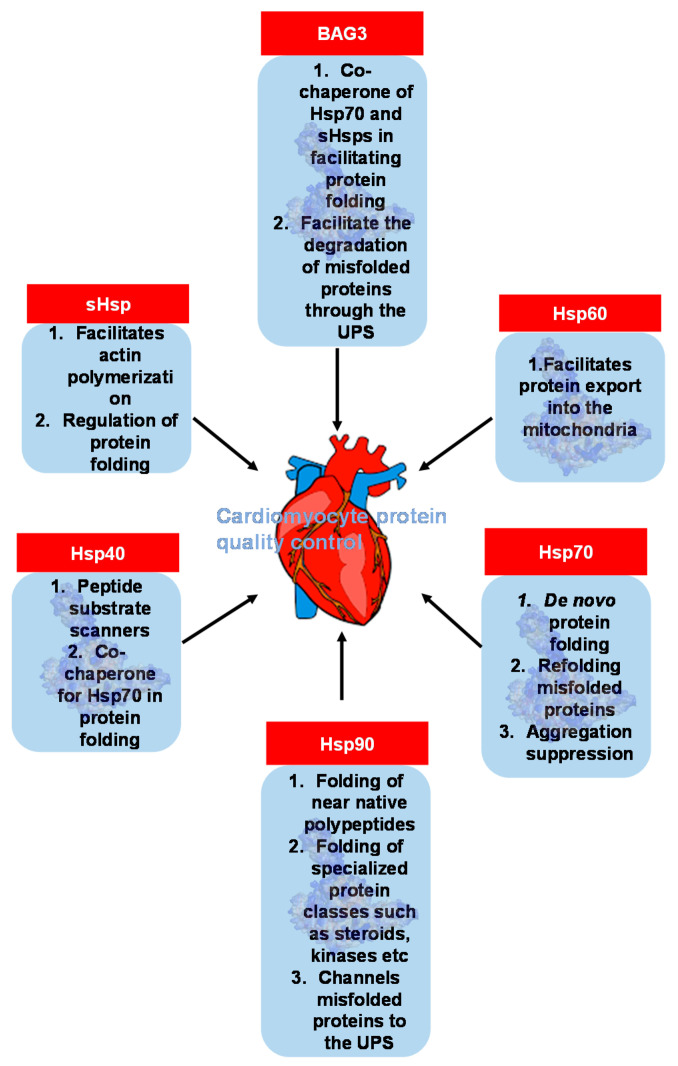
Functions of molecular chaperones in regulating cardiomyocyte protein quality. The major molecular chaperone families and their roles in the maintenance of cardiomyocyte protein quality control are illustrated. Hsp70, Hsp90, sHsp and Hsp40 are particularly important in protein folding, while Hsp60 facilitates protein translocation and BAG3 facilitates the degradation of misfolded proteins.

Hsps are a class of molecular chaperones that perform a myriad of housekeeping and stress-protective roles in cells to maintain proteostasis (Figure 5) [48]. Hsps broadly function to facilitate the correct folding and assembly of polypeptides, thus preventing the formation of aggregation-prone misfolded proteins that are toxic to cardiomyocytes [49]. Since the contractile and metabolic demands of the heart require robust protein quality control, Hsps may play crucial cardioprotective roles. Included in the Hsp family are small heat shock proteins (sHsps), which are ubiquitously expressed regulators of cellular protein folding [30]. The high expression of sHsps in the heart has been reported, where they are implicated in the maintenance of normal cardiac function and regulation of the cardiac stress response [50,51].

Genetic investigations have recently implicated Hsps in PPCM pathogenesis. Sequencing the *HSPB6* gene in DCM patients led to the identification of the rare variant p.Ser10Phe [52]. In vitro, this mutation reduced thermal resilience of the chaperone, potentially compromising its cardioprotective activity [53]. In a murine model, the p.Ser10Phe mutation-induced progressive cardiac dysfunction and HF in males, while female mice had preserved cardiac function and survival [52,54]. However, the *HSPB6* p.Ser10Phe mutation appeared to induce lethal PPCM, with a 100% fatality rate within 4 pregnancies and significant cardiac dilatation and dysfunction after 3 pregnancies [54]. This is the first description of a mutant mouse model of PPCM in which the mutation was originally discovered in humans, although *STAT3* and *Akt* transgene mice have been used previously to study the pathophysiology of PPCM [9,55]. The clinical and demographic characteristics of the p.Ser10Phe mutation carriers in the original cohort were not described, and the potential role of Hsp genes in PPCM may warrant further investigation. Although this is the first study demonstrating Hsp mutations in PPCM, genetic variation in *HSPB5*, *HSPB7* and *HSPD1* has been reported in other cardiomyopathies.

The proteostatic functions of Hsps and molecular chaperones can be hypothesised to play a crucial role in the physiological adaptation of the heart to pregnancy. Mutations in these genes may alter protein quality control systems in the heart, thereby predisposing individuals to PPCM. However, at present no other Hsp gene mutations have been described in PPCM patients to our knowledge. As such, other molecular chaperone genes such as *HSPB5* (*CRYAB*), *HSPB7* and *HSPD1* may be worth exploring for their potential as novel genetic determinants of PPCM.

*HSPB7*, sometimes referred to as the “cardiovascular Hsp” due to its high cardiac expression, is an sHsp that has attracted interest as a possible susceptibility locus for cardiomyopathy and HF [56,57]. Several genetic association studies have described protective effects of variation in *HSPB7* against DCM [29,57,58] and systolic HF [57,59,60]. These studies have been limited to individuals of European descent, and the lack of association with DCM in African Americans and Chinese populations [56,61,62] indicates that the effects of *HSPB7* variation may be population-specific, or are influenced by underlying population genetic differences. The causal relationship between *HSPB7* variation and cardiomyopathy is unclear, as many of the variants occurred in non-coding regions of the gene, did not appear to be in linkage disequilibrium with overtly protein-altering variants, and targeted sequencing of the gene in DCM patients did not yield any pathogenic variation [29,57]. However, the multiple observations of *HSPB7* polymorphisms in HF and cardiomyopathy suggests a common genetic basis for these related phenotypes. Several of these studies also reported *BAG3* variation to associate with disease risk [29,58,60], with the most recent study also demonstrating direct interaction of BAG3 protein with HspB7 [58]. These findings support the notion that *HSPB7* genetic variation may contribute to cardiomyopathy, even though the precise mechanisms are not yet known.

The *CRYAB* gene encodes αβ-crystallin or HspB5, a member of the sHsp family. In cardiomyocytes, HspB5 primarily acts to prevent the accumulation of potentially toxic protein aggregates [63], although the binding of HspB5 to TTN, actin and desmin is essential to ensure proper sarcomeric assembly and function. Mutations in *CRYAB* can trigger cardiac disease through the accumulation of protein aggregates [64]. Such a scenario has been reported in patients with cardiomyopathy, usually presenting in conjunction with skeletal myopathy and/or ocular disorders such as cataracts [64,65,66]. *CRYAB* mutations have been reported to cause isolated DCM and HCM in individuals with positive family histories for cardiomyopathy [67,68]. One of these mutations, *CRYAB* p.Arg157His, was demonstrated to impair interaction with cardiac-specific isoforms of *TTN* [67], suggesting that mutations affecting this uncharacterised domain of CRYAB may play a role in cardiomyopathy through impaired CRYAB-TTN interaction in cardiomyocytes. Given the major role of *TTN* truncations in PPCM, the effect of genetic variation in *CRYAB* may warrant further investigation.

The *HSPD1* gene encodes for a constitutively expressed Hsp60 chaperonin protein. A recent study has linked *HSPD1* mutations with cardiomyopathy when the mutation p.Thr320Ala was described in a Japanese family with DCM and arrhythmia [69]. Although the mechanisms whereby *HSPD1* may contribute to cardiomyopathy are unclear, the authors demonstrated that a similar mutation in zebrafish resulted in a DCM phenotype with mitochondrial damage and increased levels of reactive oxygen species, as well as a reduced tolerance to exercise stress [69]. Cardiac-specific deletion of the gene in mice resulted in the development of lethal DCM and HF in males, although the effects in females were not described [70]. Other *HSPD1* mutations may need to be reported in additional cardiomyopathy families before its role in disease pathogenesis is clarified.

## 5. Clinical Implications

### 5.1. Genetic Testing May Be Indicated in PPCM Patients with Family History

The identification of pathogenic cardiomyopathy-causing mutations in women with PPCM indicates that PPCM overlaps with heritable cardiomyopathy not just phenotypically, but genetically as well. In patients with genetic forms of cardiomyopathy such as DCM, ACM and HCM, current guidelines recommend the construction of a detailed family history (usually at least 3 generations), periodic clinical screening of first-degree relatives by echocardiography and electrocardiography, as well as genetic testing in the case of familial disease [71,72,73,74,75]. This approach allows at-risk family members to be identified and, when disease-causing mutations are found, cardiac follow-up can be guided by the presence of the mutation in family members. A similar strategy may be beneficial in PPCM, in which family history should be obtained from index cases and, when familial cardiovascular disease is present, genetic testing may be warranted.

Genetic techniques such as NGS allow massively parallel sequencing of vast tracts of DNA and can be used to accurately screen numerous cardiomyopathy genes in a single experiment. Such targeted sequencing panels are becoming routinely incorporated into the diagnosis of heritable cardiomyopathy in some countries [76]. However, it should be noted that there is uncertainty about whether inconclusive genetic results should be reported to patients [77]. Nevertheless, the increasing availability of NGS implies that it now may be considered more cost-effective to conduct genetic testing on asymptomatic family members than to phenotypically screen them [78,79]. In the context of familial PPCM, if a patient is found to carry a disease-causing mutation, this could allow clinical follow-up to be limited to relatives who also carry the mutation.

At this stage, it is still unclear whether individuals with mutations in any of the cardiomyopathy-causing genes require additional management during pregnancy and delivery. Limited reports have indicated that patients with DCM, HCM and ACM tend to tolerate pregnancy well, although risks of adverse cardiac events such as arrhythmias, HF, syncope and death are associated with advanced left ventricular dysfunction and prior cardiac events [80,81]. Although emergency caesarean section amongst DCM, HCM and ACM patients is rare [81], a global PPCM registry recently reported caesarean sections for up to 59% of its patients, for whom mutation carrier status was unknown [82].

### 5.2. Areas of Future Investigation

While the contribution of cardiomyopathy genes to PPCM has been demonstrated, relatively few mutations and genes have been reported so far. There is therefore still much scope for future research into the genetics of PPCM. It is likely that further screening will identify more mutations, potentially in genes that have not been described in PPCM to date, since upwards of 60 genes have been associated with DCM and other forms of cardiomyopathy [83]. Correlating genetic information with phenotypic presentation and disease outcome could have profound implications on patient management and should be explored further. This is particularly true in African populations, where the risk of PPCM is higher [4,84], the prognosis is poorer [85], and the knowledge of cardiomyopathy genetics is scarce [86]. PPCM has a broader aetiology than other forms of heritable cardiomyopathy, and further research is needed into the role of additional genes such as those involved in (auto)immunity, angiogenesis, metabolism, and oxidative stress.

Molecular chaperones and Hsp genes hold great promise as candidate genes for PPCM. In this review, several Hsp genes have been proposed as crucial genetic determinants of PPCM pathology. Recently, the mutation *HSPB6* p.Ser10Phe was demonstrated to cause PPCM in mice, indicating a role of the chaperone in protein quality control systems and stress response [54]. *BAG3* gene mutations are some of the most documented chaperone-associated mutations in cardiomyopathy. Indeed, this has led to the identification of 2 PPCM-related gene mutations in *BAG3* [32,34]. However, mutations occurring in other Hsp family genes are yet to be fully documented. Since pregnancy can induce extra stress on the heart, it is therefore plausible that Hsps may play an integral cardioprotective role in pregnant women. As such, in-depth analysis into Hsp and molecular chaperone genes may lead to the identification of novel PPCM genetic determinants that will aid in subsequent case management and drug design.

## 6. Conclusions

Recent advances have emphasised an important role of genetics in the pathophysiology of PPCM. Several studies, reviewed in this paper, suggest that up to 20% of women with PPCM have an identifiable disease-causing mutation. While a substantial overlap with heritable forms of cardiomyopathy is observed, there is much scope for future research. Given the range of physiological stresses that the body is subjected to during pregnancy, the role of Hsps in PPCM provides great potential which warrants further investigation. A better understanding of the genetics of PPCM holds the potential to improve patient management, with possible implications on treatment, prognosis, and family management.

## Figures and Tables

**Table 1:** Summary of genes associated with PPCM to date.

Gene	Molecular Function	Mutation Types in PPCM	Other Associated Disorders
*BAG3*	Co-chaperone, Z disk	Truncating	DCM, MFM
*DMD*	Sarcolemma, structure	Truncating	DCM, MD
*DSP*	Desmosome, cell–cell adhesion	Truncating	ACM, DCM, keratodermas
*FKTN*	May process dystrophin	Truncating/missense	DCM, MD
*GNB3*	G protein subunit	Association with outcome	Hypertension, night blindness
*KCNH2*	K^+^ channel, cardiac conduction	Truncating	Long QT syndrome
*LAMP2*	Lysosome, autophagy	Truncating/missense	Danon disease, DCM, HCM
*LMNA*	Nuclear lamina, structure	Truncating	DCM, MD
*MYBPC3*	Sarcomere, cardiac contraction	Missense	DCM, HCM, LVNC
*MYH6*	Sarcomere, cardiac contraction	Truncating/missense	CHD, DCM, HCM
*MYH7*	Sarcomere, cardiac contraction	Missense	DCM, HCM, LVNC, MD
*PSEN2*	May regulate APP processing	Missense	Alzheimer’s disease, DCM
*PTHLH*	Hormone	Association with risk	Brachydactyly
*RET*	Protooncogene	Missense	Multiple endocrine neoplasia
*SCN5A*	NA^+^ channel, cardiac conduction	Missense	AF, DCM, Long QT syndrome, VF
*SYNM*	Cytoskeleton	Truncating	-
*TNNC1*	Sarcomere, cardiac contraction	Missense	DCM, HCM
*TNNT2*	Sarcomere, cardiac contraction	Missense	DCM, HCM, LVNC, RCM
*TPM1*	Sarcomere, cardiac contraction	Truncating	DCM, HCM, LVNC
*TTN*	Sarcomere, cardiac contraction	Truncating	DCM, HCM, MD, MFM
*VCL*	Cytoskeleton	Truncating	DCM, HCM

ACM, arrhythmogenic cardiomyopathy; AF, atrial fibrillation; APP, amyloid precursor protein; CHD, congenital heart disease; DCM, dilated cardiomyopathy, HCM, hypertrophic cardiomyopathy; HSP, heat shock protein; LVNC, left ventricular noncompaction; MD, muscular dystrophy; MFM, myofibrillar myopathy; RCM, restrictive cardiomyopathy; VF, ventricular fibrillation.

## Data Availability

No new data were created or analysed in this study. Data sharing is not applicable to this article.
